# A conflict of visions in nutrition science: polarization, power, and the case of Ultra-Processed Foods

**DOI:** 10.3389/fpubh.2026.1748945

**Published:** 2026-03-31

**Authors:** Karen Ullian

**Affiliations:** Department of Public Health, Environments and Society, Faculty of Public Health and Policy, London School of Hygiene and Tropical Medicine, London, United Kingdom

**Keywords:** conflict of interests, nutrition sciences, polarization, science communication, Ultra-Processed Foods (UPF)

In line with wider societal trends, science has become increasingly polarized. From vaccinations to vaping, and now to UPF, disagreements are no longer side notes but dominant narratives, and any attempt at nuance is quickly dismissed, or forced into one side or another. Outlier perspectives have gained prominence, while middle-ground positions often struggle for visibility. These challenges in reaching consensus (reinforced by institutional incentives and competing interests) often delay public health decisions (1).

Polarization in science may not be a pathology but a symptom of something deeper. Using Thomas Sowell's “A Conflict of Visions” (2) as a framework helps to illuminate why certain disputes endure. Competing conceptions of human nature and social progress (Sowell's constrained and unconstrained visions) shape how scientists define research questions, interpret evidence, and propose solutions. These are not merely technical disputes about data or methods but enduring moral conundrums; though on the surface they often appear as disagreements over whether randomized controlled trials or epidemiological studies provide the best evidence for policymaking (3, 4). The distinction lies not in whether disagreement exists (5), but in how it is organized and accentuated within scientific and institutional structures (6).

The tempo and medium of scientific exchange have profoundly transformed how disagreements unfold. In an environment driven by metrics, visibility, and rapid publication cycles (7), scientific debate may take on some of the characteristics of public advocacy [another polarizing topic in itself (8, 9)]. Under pressure to deliver impact, influence policy, or shape public opinion, distinctions between evidence generation and persuasion can become blurred, contributing (often unintentionally) to polarization (10). The exchange of science-related content on social media (both by professionals and the public) has driven simplified messages, moral framing, and performative certainty; shaping how scientific ideas gain traction (11). Notably, polarization often emerges downstream of original research, as findings are simplified and amplified through media and social platforms rather than within the studies themselves. In this accelerated environment, each vision hardens its stance: one pressing for reform, the other resisting it (2). Both respond assertively, grounded in their own moral and empirical convictions, leaving little space for the reflective dialogue that complexity requires.

Nutrition science has long been shaped by tensions between decontextualized, nutrient-centric models and broader approaches that situate food within cultural, ecological, political, and social systems (12). The debate over ultra-processed foods represents the latest and most visible expression of this divide. The concept goes further from nutrients to include the food matrix and additives, while also highlighting the wider commercial determinants of health (13). Intense friction emerges between the supporters of this concept, who envisage its application in public health, and critics who argue it offers no added value beyond nutritional content. Here, Sowell's two visions are vividly at play (2).

On one side lies the unconstrained vision, which sees human nature as perfectible and systemic reform as both possible and necessary (2). From this perspective, the food system is fundamentally broken: corporations are incentivized to produce unhealthy, “quasi-addictive” products through engineering strategies reminiscent of the tobacco industry (14), and no amount of reformulation could fix a system built to profit from harm (15). What is needed, they argue, is nothing less than a systemic overhaul (16), a stance echoed in a recent high-level editorial calling for unified global responses to corporate power in food systems (17). By contrast, the constrained vision treats the limitations of human nature and policy trade-offs as unavoidable (2). In this view, progress depends on rigorous definitions and carefully designed interventions that avoid unintended harm; as exemplified in another high-profile editorial, that stresses the need for accuracy and precision to prevent regulatory overreach (18). Indeed, its proponents view the food system as deeply entrenched and argue that progress lies in making UPF healthier where possible [i.e., improving nutrient profiles (19), lowering energy density (20), and altering texture (21)] rather than attempting fundamental restructuring. Table 1 maps these contrasting visions to their manifestations within contemporary nutrition science and policy debates, particularly regarding ultra-processed foods.

**Table 1:** Mapping Sowell's visions to contemporary nutrition paradigms.

Dimension	Unconstrained vision	Constrained vision
Underlying assumption	Human systems are perfectible and reformable	Human nature and institutions resist radical change
Core problem	Food system engineered to profit from unhealthy products	Suboptimal nutrient profiles in current products
Approach to UPF	Eliminate or severely restrict	Reformulate to improve quality
Policy mechanisms	Upstream: taxation, advertising bans, procurement standards	Downstream: reformulation targets, front-of-pack labeling
Industry involvement	Corrupting influence; should be excluded from policymaking	Necessary stakeholder; requires transparent management
Evidence threshold	Precautionary (act on plausible harm)	Definitive (require causal proof)
Primary risk concern	Inaction enables continued corporate harm	Overcorrection creates unintended consequences

Most researchers and approaches fall somewhere along the continuum between these vision types rather than at the extremes; this table presents the poles for conceptual clarity.

Both visions offer valuable insights, but polarization limits opportunities for dialogue or compromise. Rather, arguments are often exchanged less to persuade than to reaffirm convictions or group belonging (22, 23). In this self-reinforcing loop, evidence is filtered through ideological lenses, and the ability to judge research on its quality, robustness, or value becomes secondary to identity and allegiance (24, 25). As a result, instead of a nuanced debate that might yield complementary and feasible solutions, a dissonant evidence-base delays much needed policies (26).

These opposing visions also shape how scientists perceive integrity and corruption within the research system. Industry funding, for instance, can be seen either as an unavoidable reality to be managed or as a moral failure demanding correction. For the unconstrained vision, any entanglement between corporate and scientific interests represents a moral violation that compromises research credibility. Industry influence is inherently corrupting; it shapes questions, distorts findings, and delays action (27), and as such, science should be free of corporate influence altogether (28). The constrained vision, by contrast, recognizes that science operates under resource limitations and that engagement with industry is often necessary (29). The goal, therefore, is to manage such relationships transparently and pragmatically, minimizing risks rather than assuming they can be eliminated (30). Depending on where one falls along this continuum of visions, industry funding may be interpreted as either a functional compromise or a breach of integrity. In practice, industry actors have repeatedly been shown to use information management strategies to protect their interests and exploit divisions within academia, often weakening regulatory pressure and contributing to permissive policy environments (20). A fragmented scientific community thus often reflects conditions shaped by industry's capacity to steer agendas and frame evidence to its advantage (20). In this sense, resolving conflicts of interest begins with acknowledging that integrity is shaped as much by moral vision and power as by procedure.

Acknowledging internal biases within academia should not obscure the broader systemic power asymmetry. Still, it is important to recognize that not all collaboration with industry is inherently compromised, and not all who criticize one's work (for instance, those who are opposed to the UPF concept) do so because of industry ties. Likewise, those who denounce corporate influence are not immune to other forms of bias (professional, ideological, or reputational) that can shape arguments as powerfully as financial sponsorship, even if they appear less problematic. Both factors deserve recognition, though they operate at different scales: structural power imbalances reflect industry's capacity to shape agendas institutionally, while cognitive biases affect how individual scientists interpret evidence. At the same time, both are reinforced by how scientific findings are selectively framed and circulated beyond their original academic context. In sum, all these factors deserve scrutiny if science is to remain credible.

So, does polarization contribute to scientific progress? At times, strong views can bring neglected issues to light, challenge assumptions, and stimulate debate. The problem, however, is not the existence of conviction itself but its unchecked dominance within scientific discourse. When strong views overshadow curiosity and nuance, they prevent balanced perspectives. Nor is disagreement itself the issue: argumentation and debate are intrinsic to science and often help refine theories (31). Yet when polarization becomes the prevailing mode of engagement, it amplifies unconscious biases and narrows the space for constructive dialogue (32). Over time, this dynamic weakens public health decision-making, leaving industry (the main beneficiary) to operate within a largely unregulated landscape (33).

And if conflicting visions are inevitable, the challenge lies in designing systems that reward openness and nuance over speed and certainty. This requires that scientists remain alert to their own passionate views, recognizing when conviction becomes overinvestment and when reasoning gives way to intellectualization [a defense mechanism that rebuts arguments with abstraction (34)]. Honest, collegial critique can be invaluable in revealing moments when such slippage occurs. Equally, work that explicitly engages with heterogeneity, uncertainty, or interdisciplinary perspectives can help sustain this kind of reflective middle ground, even in highly contested fields [see, for example ref. (35)]. Structured formats for genuine dialogue, such as paired commentaries can help sustain engagement across divides. Among these, adversarial collaboration (where scientists from opposing positions co-design studies and pre-specify methodologies) offers a particularly promising structural mechanism. By requiring researchers to work together from the outset this approach surfaces hidden assumptions, curbs confirmation bias, and generates evidence that is shared across opposing visions. Registered reports complement this approach by peer-reviewing methods before results exist, reducing outcome-driven design. Arguably, these mechanisms depend on a foundation of transparency that extends beyond financial conflicts of interest to include the ideological positions and assumptions that may influence how evidence is interpreted. Finally, scientific institutions, journals, and funding bodies can also play a central role in reshaping these dynamics by cultivating environments that value reflexivity as much as novelty and output. For instance, takeaway messages need not always be assertive or action-oriented; they can take the form of open questions.

Within science, it is essential to remain vigilant about our own biases and the tendency to dismiss criticism too quickly, while recognizing that these tendencies are reinforced by institutional incentives and structural power dynamics. Although broader cultural and systemic reforms are essential and may take time to materialize, one readily available step is a critical examination of our own positions. This does not mean delaying action (public health must often proceed despite incomplete evidence) but rather distinguishing between justified confidence and performative conviction. The former enables action; the latter prevents learning. Or, as Voltaire put it, “Doubt is not a pleasant condition, but certainty is absurd.” In science as in all inquiry, embracing that discomfort may be the most scientific act of all.
